# Shall the Patient Eat What He Craves?

**Published:** 1887-07

**Authors:** 


SHALL THE PATIENT EAT WHAT HE CRAVES?—Homœopathic experience answers, Yes; or, qualifying this answer, we might say the cravings of the patient are a valuable indication both for the choice of the dietary and in selecting the remedy. In most cases, we would advise one to allow the patient such articles as he really craves; if these be of such a nature as we think are absolutely injurious then great caution must be observed. Such cases are ones for which no rule can be made; they are cases in which the physician's judgment must decide. But there is another side to this question of diet; these cravings often aid greatly in selecting the remedy. Some cravings of the sick are so peculiar as to be very characteristic, and are often strongly indicative of a remedy. These desires for or aversions to articles of diet not only help us to decide questions of diet, not only help us to select the remedy, they do more, for when a remedy has been given, we know whether or no such and such things will help or will injure the patient. Some remedies are benefited and some aggravated by use of certain articles of diet.

Homœopathy, being scientific medicine, is helped and can use such questions as this, which are only a puzzle and a worry to the allopath. Our practice, being regulated by law, has a place for all these subsidiary questions; they are all useful under the law, but entirely useless outside of law.

Experience of man is exceedingly fallacious when not guided by definite law.

